# Essential updates 2022–2023: Surgical and adjuvant therapies for locally advanced colorectal cancer

**DOI:** 10.1002/ags3.12853

**Published:** 2024-08-19

**Authors:** Yoshiki Kajiwara, Hideki Ueno

**Affiliations:** ^1^ Department of Surgery National Defense Medical College Tokorozawa Japan

**Keywords:** adjuvant therapy, colorectal cancer, minimally invasive surgery, neoadjuvant therapy, surgical treatment

## Abstract

Pivotal articles that had been published between 2022 and 2023 on surgical and perioperative adjuvant treatments for locally advanced colorectal cancer (CRC) were reviewed. This review focuses on new evidence in the following areas: optimization of surgical procedures for colon cancer, including the optimal length of bowel resection and use of the no‐touch isolation technique; minimally invasive surgery for rectal cancer, such as laparoscopic transanal total mesorectal excision and robotic surgery; neoadjuvant treatments for rectal cancer, including total neoadjuvant therapy; neoadjuvant chemotherapy for colon cancer; and postoperative adjuvant chemotherapy for Stage II and III colon cancer. Although the current understanding may not enable perfect decision‐making for patients and medical professionals, ongoing advancements are expected to result in more effective personalized treatment plans, ultimately improving the prognosis and quality of life of patients.

## Graphical abstract

Pivotal articles that had been published between 2022 and 2023 on surgical and perioperative adjuvant treatments for locally advanced colorectal cancer (CRC) were reviewed. This review focuses on new evidence in the following areas: optimization of surgical procedures for colon cancer, minimally invasive surgery for rectal cancer, neoadjuvant treatments for colorectal cancer, and postoperative adjuvant chemotherapy for Stage II and III colon cancer.

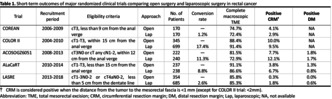

## 1 INTRODUCTION

Worldwide, more than 1.9 million new cases of colorectal cancer (CRC) and 904 000 deaths owing to CRC, making it the second leading cause of cancer death, were estimated to occur in 2022.^1^ Surgery remains the primary treatment for CRC, because many locally advanced and some resectable metastatic CRC cases can be cured by surgical intervention. However, surgical techniques for CRC have not yet been fully developed. There are variations in the conventional surgical techniques performed in different countries, often based on empirical knowledge. The rapid adoption of minimally invasive surgeries, including robotic surgery, has resulted in significant changes in CRC surgery. In addition, the development of neoadjuvant and adjuvant therapies is progressing rapidly to improve the prognosis of patients who cannot be cured by surgery alone. Therefore, the effectiveness of each treatment method must be confirmed through evidence‐based studies.

In this review article, we focus on pivotal studies published in high‐impact journals between 2022 and 2023 on surgery and perioperative treatments for locally advanced CRC. These studies have either changed or are anticipated to change the current clinical practices in colorectal cancer treatment.

## 2 RECENT EVIDENCE OF SURGICAL TREATMENT IN COLORECTAL CANCER

### 2.1 Optimal length of bowel resection

The data on the optimal length of bowel resection in CRC from a multi‐institutional study were presented by Ueno et al. under the framework of the project study group of the Japanese Society for Cancer of the Colon and Rectum. They prospectively assessed the distance of metastatic lymph nodes (LN) from the primary tumor in 2996 patients with Stage I–III colon cancer using intraoperative in vivo measurement.^2^ Only 0.1% of the patients had pericolic LN metastasis beyond 10 cm from the tumor, and these cases showed poor prognosis. Such distant pericolic spread is believed to be a systemic disease rather than a local disorder. Furthermore, although the mean length of bowel resection was approximately 11–12 cm on both the proximal and distal sides, none of the patients developed recurrence in the remaining pericolic LNs. Therefore, they concluded the “10 cm rule” to be a valid and practical criterion to define “regional” pericolic nodes.^2^

The optimal length of bowel resection should be decided according to “regional” pericolic LNs, which have potential risk of metastasis. However, there is no globally standardized definition for regional pericolic LNs. In European countries, the extent of bowel resection is believed to depend on the removal of the colon's arterial supply, and wide bowel resection is routinely employed in colon cancer surgery, such as complete mesocolic excision with central vascular ligation.^3^, ^4^ Meanwhile, the American Society of Colon and Rectal Surgeon guidelines recommend colon resection margins of 5–7 cm on the proximal and distal sides.^5^ In Japan, the “10 cm rule” had been used to determine the criterion of regional pericolic LNs since the first edition of the Japanese staging manual for colorectal cancer was published in 1977. In exceptional cases, in patients who had no feeding arteries within 10 cm of the tumor, all LNs located between the tumor and primary feeding artery were treated as regional LNs^6^. Alternative criteria, which are feeding artery‐oriented rules, have been adopted in Japan since 2006.^7^ Ueno et al. also evaluated the anatomical association with the feeding artery location. The distribution of the feeding arteries, which were located in the pericolic region within 10 cm of the primary tumor in 99.8% of the patients, did not significantly affect the status of pericolic lymph node metastasis.^2^

However, these findings were based only on the Japanese population. To evaluate whether the “10 cm rule” can be generalized in patients with colon cancer in other countries, international prospective observational cohort study for optimal bowel resection extent and central radicality for colon cancer (T‐REX study) has been conducted in the seven countries, and its results will be disclosed in the coming days. This study is expected to establish solid international evidence regarding the optimal bowel resection length for colon cancer.

### 2.2 No‐touch isolation technique

The long‐term outcomes of the no‐touch isolation technique in colon cancer surgeries have been reported. Takii et al. conducted the JCOG1006 study, a nationwide large‐scale randomized controlled trial (RCT), to evaluate whether the no‐touch method was superior to the conventional method in patients with cT3/T4 colon cancer. In conventional open colon surgery, the tumor‐bearing colon segment is mobilized before central vascular ligation (CVL). In contrast, CVL is performed before mobilization using the no‐touch method with the expectation of reducing the risk of cancer cells spreading to the liver and other organs.^8^ Two retrospective studies indicated that this method was associated with a better prognosis.^9^, ^10^ In contrast, a small‐scale RCT with approximately 230 patients showed that the no‐touch method was not associated with an improvement in prognosis.^11^ To resolve this controversy, 853 patients in the JCOG1006 study were randomized into the conventional or no‐touch groups. The findings of this study indicated that no‐touch surgery was not superior to conventional surgery in patients who underwent open surgery for colon cancer in terms of disease‐free survival (DFS), overall survival (OS), relapse‐free survival (RFS), and liver relapse‐free survival.^12^ These results provide a certain conclusion regarding the operative procedure for open surgery for colon cancer. The medial‐to‐lateral approach (MA) is the standard in recent laparoscopic right hemicolectomy and is similar to the no‐touch method.^13^, ^14^ On the other hand, the inferior approach (IA) is initiated with peritoneal dissection between the mesoileum and retroperitoneum and dissection between the mesocolon and retroperitoneum with colonic mobilization, followed by vessel dissection.^15^ Recently, a retrospective study that showed similar oncological outcomes for MA and IA in laparoscopic colectomy with complete mesocolic excision for right‐sided colon cancer was reported by Hiyoshi et al.^16^ The JCOG1006 might have provided evidence supporting the oncological safety of IA in laparoscopic surgery.

### 2.3 Laparoscopic surgery

In recent years, the effectiveness of minimally invasive surgery has been increasingly demonstrated using real‐world data. In a large‐scale registry study involving over 16 000 patients, laparoscopic surgery for CRC was linked to reduced requirements for home care and a higher likelihood of spending more time at home in the 5 years after surgery than open surgery.^17^

There are some RCTs published between 2022 and 2023 on laparoscopic surgery for rectal cancer. Jiang et al. reported the short‐term outcomes of a new large‐scale RCT (LASRE trial) involving 1039 patients, which compared the surgical quality between laparoscopic and open surgeries. In this RCT, laparoscopic surgery showed similar rates of complete total mesorectal excision to that in open surgery (85.3% and 85.8%, respectively). The rates of negative circumferential resection margins (CRM; 98.2% vs. 99.7%) and distal resection margins (99.4% vs. 100%) were also similar between laparoscopic and open surgeries, with laparoscopic surgery demonstrating a higher rate of sphincter preservation and favorable postoperative recovery.^18^ Previous RCTs which compared laparoscopic and open surgeries for rectal cancer have shown conflicting results (Table 1). The COREAN and COLOR II trials showed similar rates of negative CRM between laparoscopic and open surgeries.^19^, ^20^ However, in the ACOSOGZ6051 and ALaCaRT trials, the non‐inferiority of laparoscopic surgery over open surgery for successful resection was not established.^21^, ^22^ Jiang et al. demonstrated certain conclusions regarding this issue in an RCT that included a relatively large number of cases.

**TABLE 1:** Short‐term outcomes of major randomized clinical trials comparing open surgery and laparoscopic surgery in rectal cancer.

Trial	Recruitment period	Eligibility criteria	Approach	No. of patients	Conversion rate	Complete macroscopic TME	Positive CRM^a^	Positive DM
COREAN	2006–2009	cT3, less than 9 cm from the anal verge	Open	170	―	74.7%	4.1%	NA
			Lap	170	1.2%	72.4%	2.9%	NA
COLOR II	2004–2010	cT1‐T3, within 15 cm from the anal verge	Open	345	―	88.4%	10.0%	NA
			Lap	699	17.4%	91.4%	9.5%	NA
ACOSOGZ6051	2008–2013	cT3N0 or cT any cN1‐2, within 12 cm from the anal verge	Open	222	―	81.5%	7.7%	1.8%
			Lap	240	11.3%	72.9%	12.1%	1.7%
ALaCaRT	2010–2014	cT1‐T3, less than 15 cm from the anal verge	Open	237	―	91.1%	3.8%	1.3%
			Lap	238	8.8%	86.6%	6.7%	0.8%
LASRE	2013–2018	cT1‐3 N0‐2 or cT4aN0–2, less than 5 cm from the dentate line	Open	354	―	85.8%	0.3%	0.0%
			Lap	685	2.6%	85.3%	1.8%	0.6%

Abbreviations: CRM, circumferential resection margin; DM, distal resection margin; Lap, laparoscopic; NA, not available; TME, total mesorectal excision.

^a^ CRM is considered positive when the distance from the tumor to the mesorectal fascia is <1 mm (except for COLOR II trial: <2 mm).

### 2.4 Transanal total mesorectal excision

The short‐term outcomes of a multicenter RCT (TaLaR trial) comparing laparoscopic total mesorectal excision (LaTME) with transanal total mesorectal resection (TaTME) was reported by Liu et al in 2023.^23^ A total of 1115 patients were randomized in a 1:1 ratio to receive either TaTME or LaTME. There were no significant differences in the intraoperative complications or postoperative morbidities between the TaTME and LaTME groups. Successful resection rates were similar between the TaTME and LaTME groups (98.9% and 98.7%, respectively).^23^ The technique of TaTME with the “bottom‐to‐up” and “inside‐to‐outside” approach was reported for the first time by Sylla et al. in 2010.^24^ Since then, the use of TaTME has rapidly spread as a procedure with the potential to improve the quality of radical resection, particularly in patients with lower rectal cancer, bulky tumors, obesity, or a narrow pelvis. Although several RCTs have been conducted to compare TaTME with LaTME, these were relatively small size trials^25^, ^26^, ^27^, ^28^, ^29^ (Table 2). The TaLaR trial was the first large‐scale RCT with sufficient statistical power. This trial demonstrated that TaTME performed by skilled surgeons in selected patients with rectal cancer can achieve surgical safety and pathological outcomes similar to those of LaTME. Similar large‐scale RCTs (COLLOR III and ETAP‐GRECCAR 11) are currently ongoing.^30^, ^31^ Declaration of the long‐term outcomes of these trials is eagerly awaited to determine the appropriate indications for TaTME.

**TABLE 2:** Short‐term outcomes of recent randomized clinical trials comparing transanal versus laparoscopic total mesorectal excision.

Author	Recruitment period	Eligibility criteria	Approach	No. of patients	Conversion to open surgery	Complete macroscopic TME	Positive CRM^a^	Positive DM
Denost et al.	2008–2010	cT any, less than 6 cm from the anal verge	TaTME	50	4.0%	70.0%	4.0%*	2.0%
			LaTME	50	10.0%	62.0%	18.0%	8.0%
Zeng et al.	2016–2018	cT3N0 or cTanyN1‐2, below the peritoneal refection which is determined by MRI	TaTME	128	0.0%	94.5%	1.6%	0.0%
			LaTME	133	0.0%	89.5%	1.5%	1.5%
Ren et al.	2017–2019	cT any, less than 7 cm from the anal verge	TaTME	32	0.0%	81.3%	3.2%	0.0%
			LaTME	32	6.3%	65.6%	14.3%	6.7%
Serra‐Aracil et al. (Ta‐LaTME trial)	2015–2021	cT1–3 N0–1, less than 10 cm from the anal verge	TaTME	55	1.8%**	100%*	0.0%	NA
			LaTME	50	6.1%	88.0%	2.4%	NA
Liu et al. (TaLaR trial)	2016–2021	cT any, below the peritoneal refection based on preoperative imaging	TaTME	544	0.0%	89.5%	0.9%	0.4%
			LaTME	545	1.1%	86.1%	0.9%	0.7%

Abbreviations: CRM, circumferential resection margin; DM, distal resection margin; LapTME, laparoscopic total mesorectal excision; NA, not available; TaTME, transanal total mesorectal excision; TME, total mesorectal excision.

^a^ CRM is considered positive when the distance from the tumor to the mesorectal fascia is <1 mm.

*p < 0.05, **p < 0.001.

### 2.5 Robotic surgery

Robotic surgery for rectal cancer may provide technical advantages, especially in challenging scenarios, such as dissection in a deep and narrow pelvis. The robotic digital platform enhances the surgical precision through stable three‐dimensional vision, flexible robotic arms, steady camera movement, and motion‐scaling capabilities. A nationwide retrospective cohort study in Japan showed that patients treated with robotic surgery for rectal cancer experienced significantly lower rates of conversion to open surgery, in‐hospital mortality, intraoperative blood loss, and length of postoperative hospital stay than those who underwent laparoscopic low anterior resection.^32^ However, no significant advantage of robotic surgery was observed with regard to short‐term oncological outcomes in the ROLARR trial, which is one of the representative RCTs for robotic rectal surgery.^33^

The results of two large‐scale RCTs on robotic surgery for rectal cancer were newly disclosed between 2022 and 2023 (Table 3). Feng et al. reported the short‐term results of the REAL trial in 2022,^34^ which is a secondary endpoint aimed to assess whether robotic surgery for middle or low rectal cancer yields better surgical outcomes than laparoscopic surgery. In total, 1240 patients were enrolled and randomly assigned in a 1:1 ratio to undergo either robotic or laparoscopic surgeries. In this study, a positive CRM was observed in 4.0% of patients in the robotic surgery group, which was significantly lower than the 7.2% observed in the laparoscopic group. Additionally, the robotic surgery group exhibited significantly better postoperative gastrointestinal recovery, shorter hospital stays, fewer abdominoperineal resections, fewer conversions to open surgery, less estimated blood loss, and fewer intra‐ and postoperative complications than the laparoscopic group. The primary long‐term outcomes of this trial will be published in the future.

**TABLE 3:** Short‐term outcomes of major randomized clinical trials comparing robotic versus laparoscopic surgery for rectal cancer.

Variable	ROLARR		REAL		COLRAR	
	Ro‐TME	Lap‐TME	Ro‐TME	Lap‐TME	Ro‐TME	Lap‐TME
	(*n* = 236)	(*n* = 230)	(*n* = 586)	(*n* = 585)	(*n* = 151)	(*n* = 144)
Recruitment period	2011–2014		2016–2020		2011–2016	
Conversion to open surgery	8.1%	12.2%	1.7%	3.9%	0.7%	1.4%
Complete macroscopic TME	76.4%	77.6%	95.4%*	91.8%	80.7%	77.1%
Positive CRM^a^	5.1%	6.3%	4.0%	7.2%	4.8%	6.2%
Positive DM	NA	NA	0.4%	0.7%	NA	NA
Operative time (min, mean^b^)	298.5	261.0	173	170	264.8**	218.3
Intraoperative complication	14.8%	14.5%	5.5%*	8.7%	3.3%	3.5%
Postoperative complication within 30 d of operation	31.7%	33.1%	16.2%**^c^	23.1%^c^	NA	NA
Mortality within 30 days of operation	0.8%	0.9%	0.2%	0.2%	NA	NA

Abbreviations: CRM, circumferential resection margin; DM, distal resection margin; Lap, laparoscopic; Lap‐TME, laparoscopic total mesorectal excision; NA, not available; Ro‐TME, robotic‐assisted total mesorectal excision; TME, total mesorectal excision.

^a^ CRM is considered positive when the distance from the tumor to the mesorectal fascia is <1 mm.

^b^ Median in the REAL trial.

^c^ Complications of Clavien–Dindo grade II or higher grade.

*p < 0.05, **p < 0.001.

Another RCT on robotic rectal surgery was the COLRAR trial, and short‐term outcomes were reported by Park et al. in 2023.^35^ A total of 295 patients were enrolled and randomly assigned to the robot‐assisted TME group or the laparoscopy‐assisted TME groups. Although it has been hypothesized that robotic surgery would increase the complete TME rate by more than 10%, no statistically significant difference was observed. In the intention‐to‐treat analysis, complete TME rates were 80.7% and 77.1% in the robotic and laparoscopic surgery groups, respectively. In addition, there was no difference in postoperative complication rates between the groups. Park et al. concluded that robotic TME did not enhance the quality of TME compared with laparoscopic TME performed by experienced surgeons. However, the COLRAR trial, which originally intended to include 540 cases, was terminated prematurely because of poor accrual of cases; consequently, the statistical power of this trial might be insufficient to prove their conclusion. The long‐term outcomes of these RCTs are awaited to confirm the usefulness of robotic surgery for rectal cancer.

## 3 RECENT EVIDENCE OF NEOADJUVANT THERAPY

### 3.1 Total neoadjuvant therapy for rectal cancer

Total neoadjuvant therapy (TNT) is a treatment strategy for rectal cancer wherein the administration of preoperative chemotherapy and radiotherapy is considered to enhance tumor regression, diminish distant metastases, and ultimately enhance survival.^36^ TNT has been positioned as a standard treatment for T3 or deeper lower rectal cancer in the National Comprehensive Cancer Network (NCCN) Guidelines,^37^ with several recent studies reporting its superiority over neoadjuvant chemoradiotherapy (CRT). In 2022–2023, the results of two representative RCTs on TNT with short‐course radiotherapy will be disclosed (Table 4).

**TABLE 4:** Outcomes of major randomized clinical trials comparing TNT versus conventional CRT for rectal cancer.

Variable	Polish II		RAPIDO		PRODIGE 23		STELLAR	
	TNT	CRT	TNT	CRT	TNT	CRT	TNT	CRT
	(*n* = 256)	(*n* = 259)	(*n* = 462)	(*n* = 450)	(*n* = 231)	(n = 230)	(*n* = 298)	(*n* = 293)
Recruitment period	2008–2014		2011–2016		2012–2017		2015–2018	
Eligibility criteria	cT4 or fixed cT3		cT4, N2, lateral LN metastasis, EMVI+ or MRF + less than 16 cm from the anal verge		cT3‐4 within 15 cm from the anal verge		cT3‐4 or N1 tumor location in the distal or middle third of the rectum	
Radiotherapy	5Gy/5Fr	1.8Gy/28Fr	5Gy/5Fr	1.8Gy/28Fr or 2.0Gy/25Fr	2Gy/25Fr	2Gy/25Fr	5Gy/5Fr	2Gy/25Fr
Preoperative chemotherapy	FOLFOX4 (3 cycles)	Others^a^	CAPOX (6 cycles) or FOLFOX4 (9 cycles)	Cape	FOLFIRINOX (6 cycles) and Cape with radiotherapy	Cape	CAPOX (4 cycles)	Cape
Postoperative chemotherapy	―	―	―	CAPOX (8 cycles) or FOLFOX4 (12 cycles)	mFOLFOX6 (6 cycles) or Cape (4 cycles)	mFOLFOX6 (12 cycles) or Cape (8 cycles)	CAPOX (2 cycles)	CAPOX (6 cycles)
Toxicity (≥Grade 3)	23%	21%	48%	25%	47%	36%	27%	13%
pCR	17%	12%	28%**	14%	28%**	12%	17%	14%
3‐year OS	73%*	65%	89%	89%	91%	88%	86%*	75%
5‐year OS	NA	NA	82%	80%	NA	NA	NA	NA
8‐year OS	49%	49%	NA	NA	NA	NA	NA	NA
3‐year DFS	53%	52%	24%^b^*	30%^b^*	76%*	69%	64%	32%
8‐year DFS	43%	41%	NA	NA	NA	NA	NA	NA
3‐year local failure	22%	21%	8%	6%	4%	6%	8%	11%
5‐year local failure	NA	NA	12%	8%	NA	NA	NA	NA

Abbreviations: Cape, capecitabine; CRT, chemoradiotherapy; DFS, disease‐free survival; EMVI, extramural vascular invasion; LN, lymph node; MRF, involved mesorectal fascia; NA, not available; OS, overall survival; pCR, Pathological complete response; TNT, total neoadjuvant therapy.

^a^ Chemotherapy regimen: two 5‐day cycles of bolus 5‐Fu 325 mg/m^2^/day and leucovorin 20 mg/m^2^/day during the first and fifth week of irradiation along with five infusions of oxaliplatin 50 mg/m^2^ once weekly.

^b^ 3‐year disease‐related treatment failure.

*p < 0.05, **p < 0.0001.

The short‐term results of the RAPID trial, an international RCT assessing the effectiveness of TNT comprising a short course of radiotherapy followed by 18 weeks of systemic chemotherapy before surgery, will be reported in 2021. The TNT group (462 patients) exhibited a significantly lower rate of disease‐related treatment failure, defined as the first occurrence of locoregional failure, distant metastasis, new primary colorectal tumor, or treatment‐related death—3 years after randomization (23.7%), compared to the 450 patients who underwent standard long‐course neoadjuvant CRT followed by optional chemotherapy after surgery (30.4%), which was primarily attributed to fewer occurrences of distant metastases.^36^ In 2023, Dijkstra et al. reported 5‐year follow‐up data on locoregional control in the RAPIDO trial.^38^ Although the reduction in disease‐related treatment failure and distant metastases persisted after 5 years, the local recurrence rate following R0 or R1 resection in the TNT group (10.2%) was significantly higher than that in the conventional CRT group (6.1%). One of the reasons why TNT was associated with an increased risk of local recurrence was the difference in radiation techniques. Patients in the TNT group were more often treated with three‐dimensional‐conformed radiotherapy. Another reason could be the lack of lateral lymph node dissection. Enlarged lateral lymph nodes (ELLN) were identified as independent risk factors for local recurrence in the TNT group in the RAPIDO trial. Neoadjuvant CRT with TME alone may not be sufficient for patients with ELLN.^39^ Therefore, Dijkstra et al. suggested that if ELLN dissection had been implemented in the PAPIDO trial, the locoregional recurrence rate could potentially have been lower. The JCOG2207 (jRCTs031230415) study, a phase III RCT evaluating TNT followed by TME and selective lateral lymph node dissection for low rectal cancer, is currently ongoing in Japan. The results of this study are likely to address this issue.

The results of the STELLAR trial, a multicenter RCT conducted in China comparing short‐course radiotherapy plus neoadjuvant chemotherapy (NAC) with conventional CRT followed by surgery and adjuvant chemotherapy for locally advanced rectal cancer, were reported by Jin et al. in 2022.^40^ TNT demonstrated efficacy with acceptable levels of toxicity and could serve as an alternative to CRT for the treatment of locally advanced rectal cancer. At a median follow‐up of 35 months, the 3‐year DFS rates in the TNT and CRT groups were 64.5% and 62.3%, respectively. Although TNT resulted in approximately twice the rate of severe toxicity (≥ grade 3) compared to that in conventional CRT (26.5% vs. 12.6%), the combined rate of complete pathological response (pCR) and sustained clinical complete response (cCR) in the TNT group was 21.8%, significantly surpassing that in the conventional CRT group (12.3%). There were no significant differences in distant metastasis or local recurrence between the two groups. However, a higher 3‐year OS rate was observed in the TNT group than that in the conventional CRT group (86.5% vs. 75.1%). This study indicates the potential of TNT to improve not only local control, but also long‐term prognosis. In contrast, the 5‐year OS rates (median follow‐up, 66 months) in the PAPIDO trial were similar between the TNT (81.7%) and conventional CRT groups (80.2%).^38^ Moreover, a registry‐based study involving over 8500 patients in the United States showed that TNT was not associated with improved OS compared to that with conventional CRT, despite TNT demonstrating a significantly higher pCR rate.^41^ Furthermore, although there was an observed improvement in the 3‐year overall survival (OS) with TNT compared to CRT, the OS benefit disappeared in the Polish II trial after a long‐term follow‐up of 8 years^42^ (Table 4). Therefore, a follow‐up period longer than 5 years may be necessary to determine the long‐term benefits of TNT on clinical outcomes, particularly OS.

### 3.2 Neoadjuvant CRT for rectal cancer

In 2023, Schrag et al. reported the results of the PROSPECT trial, which had a significant impact on the preoperative treatment of rectal cancer.^43^ They conducted an international randomized trial comparing neoadjuvant FOLFOX (*n* = 585) with CRT (*n* = 543) and demonstrated the non‐inferiority of preoperative chemotherapy in terms of DFS. However, it is important to note that this trial only included patients with node‐positive cT2 or cT3 tumors. Moreover, selective CRT was administered to patients in the FOLFOX group if the primary tumor decreased in size by less than 20% or if FOLFOX was discontinued due to side effects. Similar results demonstrating the non‐inferiority of neoadjuvant FOLFOX were reported in the Chinese FOWARC trial, in which >30% of the cases were classified as cT4.^44^, ^45^ Additionally, Mei et al. reported short‐term results of the CONVERT trial in which neoadjuvant therapy with CAPOX achieved similar rates of pCR and downstaging, with a lower incidence of perioperative distant metastasis and a reduced need for preventive ileostomy than neoadjuvant CRT.^46^ A discussion on the long‐term results of this clinical trial is warranted. Based on these results, starting in 2023, the NCCN guidelines have begun to recommend NAC as an option other than TNT for T3 or deeper rectal cancer, with the addition of radiation therapy only if >20% downsizing is not achieved.^37^

Upfront surgery is also being considered for the treatment of locally advanced rectal cancer. Ruppert et al. conducted a large‐scale prospective cohort study (the OCUM study) where patients with a minimal distance between the tumor and mesorectal fascia >1 mm on high‐resolution MRI (considered low‐risk) underwent up‐front TME, while those with a distance of ≤1 mm and/or with cT4 and cT3 tumors in the lower third rectum (considered high‐risk) received CRT followed by TME.^47^ A total of 884 rectal cancer patients (cT2‐4, any cN, cM0) were treated according to this protocol, with 60% of them classified as low‐risk and undergoing upfront surgery. In the low‐risk group, the 5‐year locoregional recurrence and distant metastasis were 2.9% and 15.9%, respectively. Conversely, in the high‐risk group, the 5‐year locoregional recurrence rate was slightly higher (5.7%), but the incidence of distant metastases (30.5%) was twice as high as that of the low‐risk group. Consequently, the researchers concluded that these findings support avoiding neoadjuvant CRT in low‐risk patients and suggest intensifying neoadjuvant therapy in high‐risk patients to improve prognosis.^47^ In a retrospective cohort study, similar findings were reported by Lord et al. They suggested that high‐resolution MRI assists in identifying patients with favorable outcomes who may not require radiotherapy, provided that they lack certain MRI‐detected conditions, such as extramural venous invasion, tumor deposits, and CRM involvement.^48^ Based on these results, the 2023 NCCN guidelines recommend surgery alone as an appropriate treatment option for adequately staged, low‐risk, upper‐rectal T3 and N0 tumors.^37^ Consequently, even in Western countries, uniform preoperative CRT for locally advanced rectal cancer has been reassessed.

### 3.3 Neoadjuvant chemotherapy for colon cancer

In 2023, mature results of RCT on NAC for colon cancer (FOxTROT trial) were published.^49^ This trial included 1053 patients diagnosed with stage cT3‐4, cN0‐2, cM0 colon cancer. The patients were randomly divided into two groups: the NAC group, which received 6 weeks of FOLFOX as NAC, followed by 18 weeks of postoperative adjuvant chemotherapy; and the control group, which received 24 weeks of postoperative adjuvant chemotherapy. The ratio of the patients assigned to each group was 2:1. Additionally, in the NAC group, patients with RAS‐wild‐type tumors were randomly assigned to receive panitumumab during the first 6 weeks of NAC at a 1:1 ratio. Although panitumumab did not enhance the benefits of NAC, the NAC group exhibited significant histopathological downstaging, fewer incomplete resections, and improved 2‐year disease control. Moreover, histological regression following NAC is a robust predictor for postoperative recurrence risk and has the potential to guide postoperative therapy.^49^ These results suggest that preoperative NAC is a promising treatment option for locally advanced colon cancer. However, NAC is not necessary for all T3 colon cancer cases. Further investigation and validation are required before adopting this as the preferred treatment standard. For severe high‐risk colon cancer (cT4b/cN+ or cT3‐4a/cN2‐3), the JCOG 2006 (jRCTs031210365) trial, a randomized phase II trial comparing preoperative chemotherapy with mFOLFOX6 versus FOLFOXIRI, is currently underway. This clinical trial offers new evidence regarding the appropriate use of NAC in the treatment of colon cancer.

## 4 RECENT EVIDENCE OF POSTOPERATIVE ADJUVANT CHEMOTHERAPY

### 4.1 Adjuvant chemotherapy for Stage III colon cancer

Six months has been the standard duration of oxaliplatin‐based adjuvant chemotherapy (FOLFOX or CAPOX) for patients with Stage III colon cancer.^50^, ^51^, ^52^, ^53^, ^54^ However, almost 90% of patients with CRC treated with oxaliplatin for 6 months develop chemotherapy‐induced peripheral neuropathy. Lee et al. reported that the risk factors for oxaliplatin‐induced peripheral neuropathy in an adjuvant setting include lower physical activity, higher BMI, diabetes, and a longer planned treatment duration.^55^

To evaluate the non‐inferiority of 3 months of FOLFOX or CAPOX therapy compared to 6 months therapy in terms of the rate of DFS at 3 years, the International Duration Evaluation of Adjuvant Therapy (IDEA) collaboration was formed. Within the IDEA collaboration, data from six RCTs involving patients with Stage III colon cancer were pooled and analyzed.^56^, ^57^ In this collaborative study, the noninferiority of 3 months versus 6 months of treatment was not confirmed in the overall study population for DFS and OS. However, 3 months of CAPOX was not inferior to 6 months of CAPOX in terms of DFS in patients with low‐risk colon cancer (T1‐3N1). Conversely, in the subgroup of patients with high‐risk colon cancer (T4 and/or N2) treated with FOLFOX for 3 months, DFS was reduced compared to that in the subgroup randomized to 6 months of therapy. In 2022, similar long‐term results were reported in RCTs with Asian cohorts (ACHIEVE and KCOG CO09‐07).^58^, ^59^ Following collaboration with the IDEA, the NCCN guidelines suggested a 3‐month CAPOX regimen for low‐risk Stage III cases and recommended considering this treatment as an optional choice for high‐risk Stage III cases.^60^

Additionally, in 2023, Gallois et al. reported the prognostic impact of early discontinuation of oxaliplatin in a 6‐month regimen for Stage III colon cancer, using pooled data from over 10 000 patients across 11 clinical trials.^61^ Early discontinuation of all treatments, defined as receiving up to 75% of the full therapy cycle, had an independent negative prognostic impact, resulting in an almost 10% decrease in DFS and OS. In contrast, the early discontinuation of oxaliplatin alone was not associated with poor outcomes. However, patients who received less than 50% of the planned oxaliplatin cycles had poorer outcomes than those who received at least 50% of the planned cycles.^61^ Therefore, it may be justified to intensively monitor the emergence of oxaliplatin‐induced peripheral neuropathy and stop oxaliplatin immediately when patients exhibit at least grade 2 neurotoxicity and have received at least 50% of the planned oxaliplatin cycles while continuing with the other drugs in the 6 months regimens.

### 4.2 Adjuvant chemotherapy for stage II colon cancer

Adjuvant chemotherapy is recommended for high‐risk patients with Stage II CRC. The IDEA collaboration study included more than 3200 high‐risk Stage II patients and showed results similar to those of the Stage III analysis. While the 3‐month regimen did not show noninferiority in DFS compared to the 6‐month regimen, the convenience, reduced toxicity, and cost of the 3‐month adjuvant CAPOX suggest that it is a potential option for high‐risk Stage II colon cancer if oxaliplatin‐based chemotherapy is appropriate.^62^, ^63^

In Stage II CRC, the high‐risk groups commonly include patients with poorly differentiated or undifferentiated histology, lymphatic or vascular invasion, bowel obstruction, fewer than 12 examined lymph nodes, perineural invasion, localized perforation, positive margins, and high‐grade tumor budding.^60^, ^64^ However, there is no definitive evidence regarding the utility of these risk factors in selecting adjuvant chemotherapy.

Recently, the presence of circulating tumor DNA (ctDNA) after surgery has drawn attention as a risk factor for CRC recurrence of CRC.^65^, ^66^ The results of the DYNAMIC trial, an RCT designed to investigate whether a ctDNA‐guided approach, compared with a standard approach in Stage II colon cancer, could reduce the use of adjuvant treatment without increasing the risk of recurrence, were reported by Tie et al. in 2022.^67^ Treating only patients with detectable ctDNA reduced the percentage of patients receiving adjuvant therapy to 15% compared to 28% with conventional management, without compromising RFS (3‐year RFS: 91.7% in the ctDNA group vs. 92.4% in the conventional group). In addition to the measurable false negative rate, the screening costs of ctDNA for all Stage II patients should be considered in clinical applications.

Histological categorization of desmoplastic reactions (DR) is a novel tool for risk stratification in CRC. DR is classified as mature, intermediate, or immature based on the presence of hyalinized collagen bundles and myxoid stroma at the invasive front of the tumor using hematoxylin and eosin staining.^68^ In a prospective multicenter study of 991 Stage II patients, a non‐mature DR pattern was identified as an independent risk factor for recurrence.^69^ Consequently, the DR pattern was used as one of the inclusion criteria, together with three other promising risk factors (pT4, high‐grade tumor budding, and perineural invasion), in the JCOG1805 (jRCTs031190186) trial, which is a phase 3 RCT of adjuvant chemotherapy for Stage II high‐risk CRC patients.

## 5 CONCLUSIONS

In this review, we have briefly covered the recent updates and trends in locally advanced CRC treatment, as reported in pivotal studies. Although the current situation is not sufficient to achieve perfect decision‐making for patients and medical professionals, future developments are expected to lead to more effective personalized treatments, thereby improving the quality of life of patients. We should always stay in touch with novel ideas and concepts as new evidence emerges.

## AUTHOR CONTRIBUTIONS

**Yoshiki Kajiwara:** Conceptualization; writing – original draft. **Hideki Ueno:** Conceptualization; supervision; writing – review and editing.

## FUNDING INFORMATION

None.

## CONFLICT OF INTEREST STATEMENT

Hideki Ueno is an editorial board member of *Annals of Gastroenterological Surgery*. The other author has no conflicts of interest to declare.

## ETHICS STATEMENT

Approval of the research protocol: N/A.

Informed consent: N/A.

Registry and the Registration No. of the study/trial: N/A.

Animal studies: N/A.
